# First use of adeno-associated viruses in the human inner ear

**DOI:** 10.1016/j.omtm.2024.101197

**Published:** 2024-02-10

**Authors:** Lukas D. Landegger

**Affiliations:** 1 Department of Otolaryngology-Head and Neck Surgery, Vienna General Hospital, Medical University of Vienna, Waehringer Guertel 18-20, 1090 Vienna, Austria; 2 Christian Doppler Laboratory for Inner Ear Research, Department of Otolaryngology-Head and Neck Surgery, Vienna General Hospital, Medical University of Vienna, Waehringer Guertel 18-20, 1090 Vienna, Austria; 3 Department of Otolaryngology-Head and Neck Surgery, Stanford University School of Medicine, 801 Welch Road, Palo Alto, CA 94305, USA

## Main text

Last year was an incredibly exciting time for inner ear gene therapy. However, the events of 2023 cannot be adequately summarized without acknowledging a pivotal moment in December 2017: the first US Food and Drug Administration approval of an *in vivo* gene therapy, voretigene neparvovec (Luxturna). The approval of Luxturna was the culmination of decades of preclinical research and preliminary testing in humans. This milestone propelled further developments for gene therapy in the retina and led to the experimental application of adeno-associated viruses (AAVs) for other ophthalmologic diseases, resulting in more than 100 clinical gene therapy trials for eye conditions.

Despite many parallels between the two sensory organs, the use of AAVs in the inner ear has not progressed as quickly. This slower progress could be attributed to the anatomical complexity of the inner ear and the difficulty of surgical access; the auditory and vestibular systems are encased together in one of the hardest bones of the human body. While vestibular implants have shown promising results in a handful of patients in small clinical trials,^1^ any novel auditory treatment for severe to profound sensorineural hearing loss will be compared to the established gold standard, the cochlear implant. As the most successful neuroprosthetic device, the cochlear implant has restored quality of life for hundreds of thousands of patients over the last decades. This device reliably stimulates the spiral ganglion neurons forming the auditory nerve according to externally detected sound frequencies and loudness. Nevertheless, there is room for improvement. Bypassing the cochlea’s sensory hair cells with electrical currents reduces frequency specificity, which is important for music perception, and results in difficulties in environments with substantial background noise. Efforts to improve upon this device include the optogenetic cochlear implant,^2^ which has shown promising results in combination with AAVs in animal models.

Currently, AAVs are primarily used to deliver genetic cargo capable of reinstating cellular function. Thus far, based on preclinical data, the most successful approaches have been established in recessive diseases,^3^ similar to the above-mentioned voretigene neparvovec for Leber congenital amaurosis. Although otologic rescue experiments were carried out in rodents, safety analyses in non-human primates were necessary prior to initiating human studies.^4^^,^^5^^,^^6^^,^^7^ These safety studies revealed no major side effects, prompting several companies and academic centers to enroll patients with otoferlin (OTOF)-related hearing loss in their respective clinical trials. OTOF deficiency leads to prelingual, severe to complete, autosomal-recessive deafness 9 (DFNB9), resulting in auditory neuropathy spectrum disorder (ANSD; i.e., defective inner hair cell synaptic transmission). Although the OTOF coding region is ∼6 kb, dual-vector AAV strategies that have worked in other organs have also been successful in OTOF knockout mice and could rescue hearing in newborn mice.^8^^,^^9^ The correct timing of the intervention is crucial, as mice are born deaf, while humans develop mature hearing organs *in utero*, i.e., an early postnatal injection in mice correlates to an *in utero* injection in humans.

Clinically, ANSD is sometimes initially overlooked, as most newborn hearing screening programs around the world employ otoacoustic emissions, which only test the function of outer hair cells. Typically, in the case of negative screening results, auditory brainstem responses (ABRs) are used to diagnose ANSD. Due to associated costs and required facilities, DNA sequencing with subsequent genetic counseling has been limited for many years, even in high-quality healthcare systems. The therapeutic relevance of DNA sequencing in patients with severe to profound hearing loss has also been questioned, as these children would be offered cochlear implants regardless of disease etiology. Nonetheless, we now know that cochlear implant performance also depends on the underlying cause of hearing loss and correlates with the respective genetic mutation. These findings combined with reduced sequencing costs and the motivation to include as many patients with OTOF as possible in each study have recently resulted in unprecedented rates of genetic testing at many centers.

While all companies focusing on OTOF and inner ear gene therapy in general have revealed some information regarding their clinical trials on their respective websites and in online registries, public insights into the different therapeutic strategies have been limited. The first results were finally revealed at the 30^th^ European Society of Gene and Cell Therapy Annual Congress in Brussels in October 2023, where an academic group led by Professor Yilai Shu from Fudan University in Shanghai presented their data on five children with DFNB9 and complete hearing loss between the ages of 2 and 6 years. AAV1-hOTOF was unilaterally injected via a minimally invasive endoscopic approach through the external auditory canal and into the cochlea via the round window membrane (9 × 10^11^ in one patient and 1.5 × 10^12^ vg for the other four). Follow-up for a minimum of 13 weeks revealed no serious adverse events, an increase in neutralizing AAV1 antibodies in the peripheral blood of all patients, and, most importantly, hearing restoration in four out of five children (32–50 dB reduction in average ABR thresholds), and improved speech perception in three out of five patients. Immediately following the presentation of these results, Decibel Therapeutics/Regeneron Pharmaceuticals also announced that a first patient under 2 years of age had been dosed with AAV1-hOTOF including their proprietary Myo15 promoter, which limits the transduction to hair cells. As observed in the other cohort, there were improved ABR thresholds without any serious adverse events. While not all details regarding the studies have been revealed, they certainly look promising and will encourage the field to develop further AAV-based therapies for the inner ear.

Compared to the earlier non-human primate experiments that required a retroauricular approach in combination with a mastoidectomy, these studies have shown that the surgery can be less invasive than expected and that efficient delivery appears to be feasible without any major complications. Even before these first results and despite the uncertainties, patients appear to be open to novel inner ear gene therapies,^10^ though it is currently unclear when these therapies will become available to them.
